# Temporal variation in sex pheromone release from individual *Chilo suppressalis* (Lepidoptera: Crambidae) females and maximization of male trapping

**DOI:** 10.1007/s44297-026-00071-w

**Published:** 2026-04-01

**Authors:** Chizhou Liang, Qianshuang Guo, Shaolong Wu, Tianbo Liu, Wei Cheng, Xiangwen Wu, Haibo Bao, Yongjun Du

**Affiliations:** 1 Zhejiang Provincial Plant Protection, Quarantine and Pesticide Management Station, Hangzhou, China; 2 NewCon Inc., Ningbo, China; 3 Hunan Provincial Tobacco Science Research Institute, Changsha, China; 4 Shanghai Agricultural Technology Extension and Service Center, Shanghai, China; 5 Institute of Pesticide and Environmental Toxicology, Zhejiang University, Hangzhou, China

**Keywords:** *Chilo suppressalis*, SPME extraction, Individual pheromone variation, Blend ratio, Behavioral resistance

## Abstract

Insects exhibit considerable variation in their sex pheromone blends, but evidence for temporal variation in individual *Chilo suppressalis* females has been lacking. Using solid-phase microextraction (SPME), we characterized the variability in the sex pheromone composition of *C. suppressalis* females and its implications for male attraction. Although the female reproductive system matures by day one, the titer and release rhythm of the major component, (*cis)*-11-hexadecenal [(*Z*)*-*11–16:Ald], showed substantial individual variation, particularly among females aged 1 to 3 days. Two-day-old females exhibited the most consistent detection frequency and the longest release window, accompanied by a significantly altered ratio of (*Z*)*-*11–16:Ald to the minor component (*cis)*-9-hexadecenal [(*Z*)*-*9–16:Ald]. A positive correlation was established between the proportion of (*Z*)*-*11–16:Ald in the blend and its absolute titer. Higher temperatures increased the release of (*Z*)*-*11–16:Ald from the pheromone gland surface, while mating drastically reduced it. Subsequent field trials demonstrated that the total dosage influenced the attractiveness of different pheromone ratios. At a low dose of 760 μg, the (*Z*)*-*11- to (*Z*)*-*9–16:Ald ratios had no significant effect, with 10:1, 16:1, and 32:1 each attracting a comparable number of males. At a medium dose of 1520 μg, the 10:1 and 16:1 ratios were most attractive, while at a higher dose of 2280 μg, the 16:1 ratio was clearly superior. Crucially, after five years of mass trapping with a fixed 10:1 blend, local populations shifted their behavioral responses: one site showed enhanced attraction to a 30:1 ratio, while another exhibited increased response to a 7:1 ratio, indicating directional adaptation. Intraspecific variation in female pheromone release and male response is substantial in *C. suppressalis*. Long-term use of a single pheromone blend can drive behavioral resistance, necessitating rotation of multiple blends or integration with complementary control tactics for sustainable pest management.

**Supplementary Information:**

The online version contains supplementary material available at 10.1007/s44297-026-00071-w.

## Introduction

Upon reaching sexual maturity, female lepidopteran insects exhibit calling behavior, releasing a species-specific blend of sex pheromones from their pheromone glands to initiate conspecific male orientation behavior for mating [1, 2]. This observed specificity is mainly governed by the complete composition in a precise ratio and dosage [3, 4]. Adult males are strongly and innately attracted to female sex pheromones, a signaling system regarded as an example of stabilizing genetic selection[5, 6]. However, this communication system is complex and is shaped by multiple biotic and abiotic variables. Pheromone production in females varies with age [7, 8], adult feeding [9], mating status [7], circadian rhythms [10, 11], host plants [8, 12], and climatic conditions [13], while sub-lethal insecticides can also induce significant individual variation [14]. Seasonal differences are a combination of host plants, climatic factors, diurnal rhythms and others. Thus, sex pheromone systems are not static, and their genetic variation and differentiation are shaped by natural selection [6, 15], which has, in turn, selected for adaptive changes in the male olfactory system [16], particularly in response to individual variations in pheromone blends [17, 18]. Multiple factors ultimately affect two key processes: the biosynthesis and production of sex pheromones in females [19, 20] and olfactory perception and behavioral response in males [21]. Variation in female pheromone output and male olfactory sensitivity can thus co-evolve, leading to mutual adaptation within the population.

*Chilo suppressalis* (Walker) (Lepidoptera: Crambidae) females produce a sex pheromone blend consisting primarily of (*cis*)−11-hexadecenal [(*Z*)*-*11–16:Ald], along with the minor components (*cis*)−9-hexadecenal (*Z*9–16:Ald), (*cis*)−13-octadecenal [(*Z*)*-*13–18:Ald], *cis*−11-hexadecen-1-ol [(*Z*)*-*11–16:OH], and hexadecanal (16:Ald) [22]. Male attraction is synergistically enhanced by the optimized ratio and dosage of (*Z*)*-*11–16:Ald, (*Z*)*-*9–16:Ald and (*Z*)*-*13–18:Ald [22]. While the inclusion of (*Z*)−11–16:OH does not lead to a significant rise in trap captures, it contributes to species specificity [22]. Although the behavioral role of 16:Ald remains undetermined, the male response is highly sensitive to variations in both the ratio and dosage of (*Z*)*-*11- and (*Z*)*-*9–16:Ald [23], making these parameters crucial for attraction in most areas [23]. Mating behavior in *C. suppressalis* is further influenced by factors such as adult age [24], light intensity and photoperiod [25], temperature and humidity [26], and seasonal variations [12]. Therefore, understanding variation in both the production (female) and recognition (male) of sex pheromones is essential for optimizing pheromone-based monitoring and control strategies.

*Chilo suppressalis* is widely distributed across a wide latitudinal range, from tropical to subtropical zones in Asia, southern Europe, and northern Africa[27]. Its range in China spans from the northeast to the southernmost regions, extending westward to Ningxia and Xinjiang. This broad distribution establishes *C. suppressalis* as a major borer pest in key rice-producing provinces, including Yunnan, Sichuan, Guizhou, Hunan, Jiangxi, Zhejiang, Anhui, and the northeastern provinces [28]. As an oligophagous species, it also infests other host plants, including wheat, rapeseed, sugarcane, corn, sorghum, and water-oat [12, 27]. The management of *C. suppressalis* has traditionally relied heavily on chemical insecticides. However, overuse of pesticides has accelerated the evolution of resistance in pest populations[29], prompting routine monitoring of resistance levels in several key provinces across China. Given this challenge, monitoring based on sex pheromone trapping has gained increasing adoption among rice growers in many regions. This method is used to determine the optimal insecticide application timing and is often integrated with mass trapping and mating disruption strategies to enhance the overall management of *C. suppressalis*.

A primary challenge in sex pheromone trapping is the geographic and individual variation in the male olfactory response, which can significantly affect the efficacy and reliability of monitoring and control [23]. It follows that similar variation may exist in the conspecific sex pheromone itself. Over the past five decades, researchers have identified the sex pheromones of more than 2,000 lepidopteran species [30]; however, temporal variation in sex pheromone composition remains poorly understood for most species, including *C. suppressalis* [5]. The objectives of this study were to determine the temporal variation in the *C. suppressalis* sex pheromone and to develop a more effective blend for field application.

## Materials and methods

### Insects

Larvae of *Chilo suppressalis* within rice straws were collected from a paddy field in Qianwei County, Sichuan Province, China (29.2081°N,103.9493°E), and transported to the laboratory in the spring. Pupae were separated from the rice straw after pupation and maintained in a climate-controlled chamber. Rearing conditions were set as follows: a photoperiod of 14 L:10D (light on at 06:00), a temperature of 22 ± 1 °C, and a relative humidity of 70 ± 10%. Newly eclosed females were collected (designated as 0 days old) and kept in a screen cage provisioned with a 10% sugar solution. Moths aged 24 h were designated as 1 day old.

### Extraction and analysis of individual female C. suppressalis sex pheromones

The solid phase micro-extraction (SPME) method followed that of reference [31] and was used for the extraction of female *C. suppressalis* sex pheromone. Under a stereo dissection microscope (SMZ172, Motic, Xiamen, China), the tip of the abdomen was gently compressed to evert the pheromone glands. An SPME fiber (100 μm PDMS/DVB, Supelco, Bellefonte, PA, USA) was then used to gently and evenly rub back and forth for 2 min on the surface of the outer cuticle of the exposed pheromone glands. This SPME adsorbing method was used to analyze the temporal variation in sex pheromone release in females during scotophase at different female ages. Prior to each extraction, the SPME fiber was activated by heating in the GC injector at 250 °C for 5 min. After retrieval into the needle, the fiber was introduced into the inlet GC, extended, and thermally desorbed. Analysis was performed on an Agilent 8860 GC-5977B MS system (Santa Clara, CA, USA) fitted with an HP-5MS capillary column (30 m × 250 μm × 0.25 μm; Agilent Technologies, Santa Clara, CA, USA). The temperature programmed in GC began at 60 °C for 1 min, ramped to 180 °C at 10 °C/min, then an increase of 3 °C/min to 210 °C, and again ramped to 250 °C at 10 °C/min, followed by a final isothermal hold at 250 °C for 10 min. The ion source was maintained at 230 °C with an electron ionization energy of 70 eV, using high-purity helium as the carrier gas.

Quantification of *C. suppressalis* sex pheromone compounds ((*Z*)*-*11–16:Ald, (*Z*)*-*9–16:Ald, 16:Ald, (*Z*)*-*11–16:OH and (*Z*)*-*13–18:Ald) was based on their individual standard curves. All pheromone standards and blends were synthesized and purified to ≥ 97% purity by NewCon Inc. (Ningbo, China).

### Age-dependent temporal variation in sex pheromone release in individual female C. suppressalis

To assess and track temporal variation in sex pheromone secretion, each individual female labeled and numbered was repeatedly sampled and analyzed every 2 h during scotophase at 0, 1, 2, and 3 days post-eclosioin. Sampling times were 18:00, 20:00, 22:00, 24:00, 02:00, and 04:00. All handling was performed gently to ensure moth survival, and the moth remained active throughout the experiment. The experiment was replicated 15 times (female moths), each with a randomly selected female moth.

### Effect of C. suppressalis body weight on sex pheromone release

*Chilo suppressalis* females were individually weighed in a 1.5 ml centrifuge tube using an electronic balance (AUW120D, SHIMADZU, Japan). Immediately after weighing, the activated SPME fiber was then used to adsorb the pheromone compounds on the surface cuticle of the pheromone gland of each female for 2 min, as described in Sect. "Extraction and analysis of individual female C. suppressalis sex pheromones". After the adsorption was completed, the fiber was retrieved into the needle, followed by immediate injection into the GC‒MS for desorption and analysis, as described in Sect. "Extraction and analysis of individual female C. suppressalis sex pheromones". A total of 61 female moths were individually weighed and extracted.

### Effect of female mating status on sex pheromone release

Ten male and ten female moths, each one day old, were paired and placed in an insect cage (a cylindrical insect rearing cage made of 120-mesh nylon mesh, 25 cm in diameter and 30 cm in height). After the onset of scotophase, mating behavior was observed and checked every other 20 min under red light. Once *C. suppressalis* females observed mating, they were gently moved to an individual and labeled with a 50-ml plastic centrifuge tube. Pheromone collection from these mated females occurred at two time points: immediately (0 h) or 24 h post-mating. Concurrently, unmated females of the same age provided control samples. All extractions adhered to the SPME protocol in Sect. "Extraction and analysis of individual female C. suppressalis sex pheromones".

### Effect of temperature on sex pheromone release

Extractions of headspace pheromone were conducted from individual female *C. suppressalis* at ambient temperatures of 15, 25, and 35 °C. Gentle pressure was applied to the female's abdomen to evert its pheromone gland. The terminal abdominal segment containing the exposed gland was then carefully positioned inside one end of a glass pipette (5 mm inner diameter, 30 mm length). A conditioned SPME fiber was then slowly introduced into the far end of the glass pipette, ensuring no direct contact with the moth’s body. After a 5-min adsorption period, the SPME fiber was introduced into the GC‒MS injector for desorption and analysis. After allowing the female moths to adsorb for 5 min at each temperature, they were transferred to the next temperature setting and left for 10 min to acclimatize to the new environment before the next headspace adsorption was conducted. We repeated this adsorption process sequentially across all temperature treatments. The experiment was replicated six times, with one female per replicate.

### Field experiments for male C. suppressalis trapping

Pheromone lures were prepared using PVC capillary tubing (0.6 mm inner diameter, 1.1 mm outer diameter and 80 mm length) (NewCon Inc., Ningbo, China). To stabilize the pheromone compounds in the lure, butylated hydroxytoluene (BHT) was added at a concentration of 3%. The mixture was then dissolved in petroleum ether (analytical reagent, boiling point 90–120 °C) at an appropriate concentration. After thoroughly stirring the mixed solution, 16 μl was slowly injected into the tubing using a 1 ml medical syringe, and both ends were heat-sealed. The finished PVC lures were sealed in aluminum foil bags, shipped to the test sites by Express mail, and stored at −20 °C until use. Modified plastic flying moth traps (PT-FMT, NewCon Inc., Ningbo, China), each baited with a single sex pheromone lure, were deployed at a height of approximately 0.8 m above the water surface in the rice paddies.

The pheromone blends for testing contained varying ratios of (*Z*)*-*11- to (*Z*)*-*9–16:Ald, while the total combined dosage of these two components was kept constant at 1080 μg in the initial tests. The following minor components were added at fixed dosages: 16:Ald(225 μg), (*Z*)*-*11–16:OH (95 μg), and (*Z*)*-*13–18:Ald (120 μg). For the subsequent experiment examining dosage and ratio, lures contained total pheromone loads of 760 μg, 1520 μg and 2280 μg.

Field trials to determine the optimal (*Z*)*-*11- to (*Z*)*-*9–16:Ald were conducted in a paddy field in Yinzhou district, Zhejiang Province (29.5213°N, 121.6571°E), from May 25 to June 7, 2022. Seven blend ratios of (*Z*)*-*11- to (*Z*)*-*9–16:Ald were tested: 7:1, 10:1, 13:1, 16:1, 32:1, 89:1, and 100:0, with a total dosage of active ingredient load of 1520 μg per lure. The amounts of (*Z*)*-*11–16:OH, 16:Ald and (*Z*)*-*13–18:Ald remained constant. The control traps contained no lure. The experimental layout consisted of a straight line of traps spaced 30 m apart. The design was a randomized complete block with six replicates, where blocks were separated by > 50 m and trap positions were randomized within each block. Traps were checked and emptied every other morning. The dosage-ratio trials were conducted from June 12 to June 21, 2022 in Tianyang County (23.8167°N, 106.8333°E), Guangxi, China.

To assess the long-term effect of mass trapping of *C. suppressalis* males with a single blend ratio of (*Z*)*-*11 to (*Z*)*-*9–16:Ald in the pheromone mixture on the future trapping capacity, field tests were conducted at two sites in Hunan Province, China: Huangtuling (27.388°N, 113.545°E, from August 28 to October 22, 2023), You County, and Sifen (27.528°N, 113.498°E, from August 29 to September 30), Liling County, Hunan Province, China. The two sites were over 200 km apart. Both locations had used lures with a 10:1 ratio for mass trapping of *C. suppressalis* during the five consecutive years prior to this experiment. In the current experiment, the following ratios were evaluated: 30:1, 16:1, 13:1, 10:1, 7:1, 4:1, and 1:1. Rice fields with no history of mass trapping served as controls. Other trapping protocols in the treatment and control fields were as described above.

### Statistical Analysis

Data analysis was conducted using SPSS [32]. Temporal changes in the amount of (*Z*)*-*11–16:Ald detected from individual females during scotophase at each age were analyzed using one-way repeated-measures ANOVA. One-way ANOVA was applied to total pheromone per age, detection frequency of (*Z*)*-*11–16:Ald per age, the (*Z*)*-*11-/(*Z*)*-*9–16:Ald ratio, and trap counts. A chi-square test assessed age-dependent differences in the proportion of females emitting detectable (*Z*)*-*11–16:Ald. Post hoc comparisons of means were performed using Duncan's multiple range test. Prior to ANOVA, data were transformed to meet normality assumptions: individual (Z)−11–16:Ald amounts were ln(x + 1) transformed, and trap counts were log(y + 1) transformed. Finally, Pearson correlation analysis examined the relationship between the pheromone ratio and the total (*Z*)*-*11–16:Ald, as well as between female body weight and the total amount of (*Z*)*-*11–16:Ald or the frequency of its detection. To assess the impact of long-term, large-scale sex pheromone trapping on individual response variation in field populations, an independent samples t-test was used to compare differences in male capture rates across different pheromone ratios. The male capture rate was calculated as the number of male moths captured by traps baited with a specific lure ratio divided by the total number of male moths captured by all traps.

## Results

### Age-dependent temporal variation in sex pheromone release in individual female C. suppressalis

**Fig. 1:**
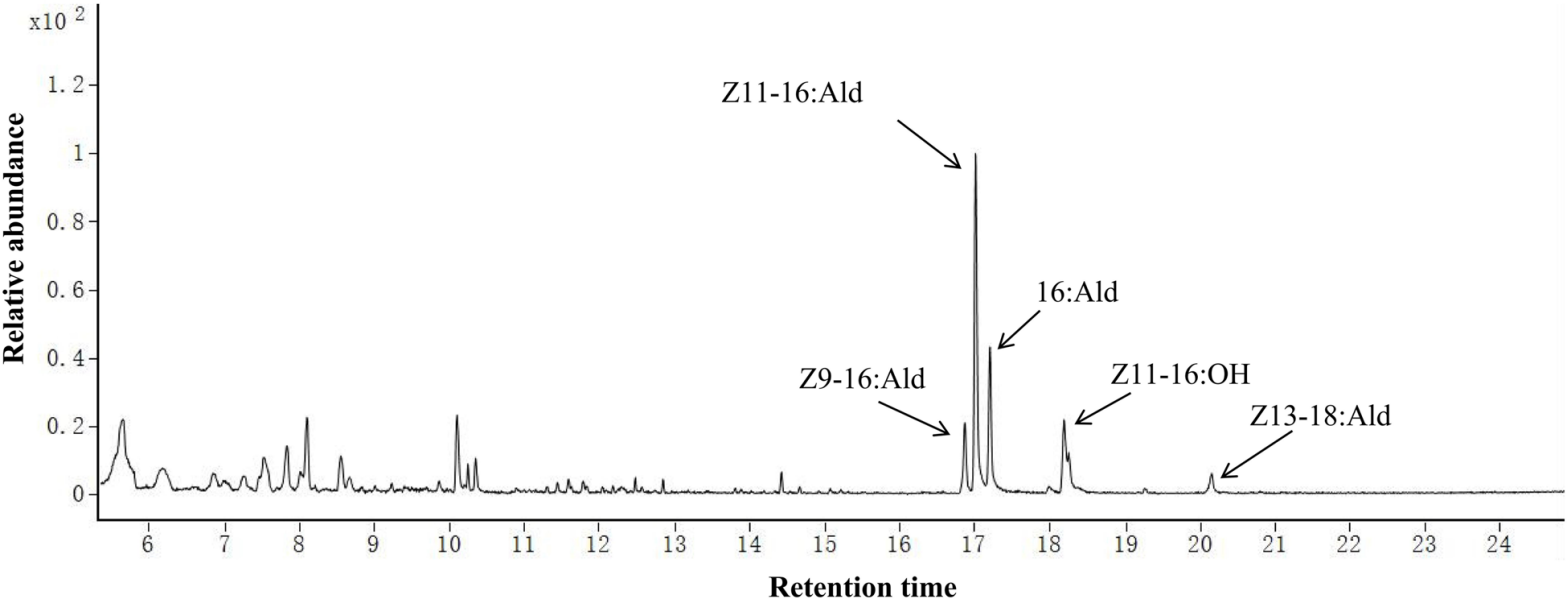
GC‒MS analysis of the pheromone mixture extracted from the outer cuticle of sex pheromone glands of female *C. suppressalis*

**Fig. 2:**
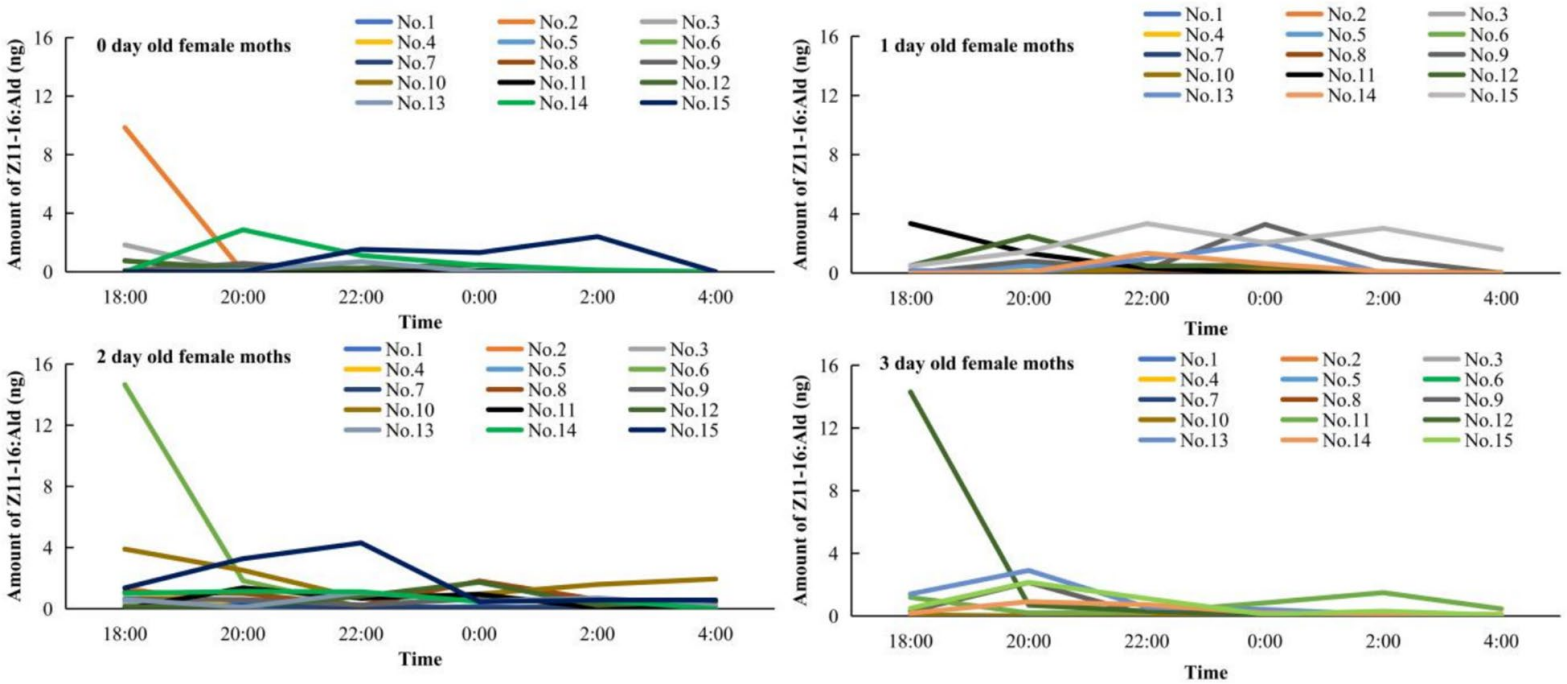
Temporal changes in *Z*11–16:Ald titers in a single female *C. suppressalis*. Different curves in the figure represent individual females and their label numbers. A, 0 d old; B, 1 d old; C, 2 d old; D, 3 d old

By the first day following eclosion, female *C. suppressalis* had a mature reproductive system with fully developed ovaries. Solid-phase microextraction (SPME) from the female pheromone gland detected five components, namely, the major component (*cis*)−11-hexadecenal [(*Z*)*-*11–16:Ald] and the minor components (*cis*)−9-hexadecenal [(*Z*)*-*9–16:Ald], hexadecanal (16:Ald), (*cis*)−11-hexadecen-1-ol [(*Z*)*-*11–16:OH], and (*cis*)−13-octadecenal [(*Z*)*-*13–18:Ald] (Fig. 1). The titer of sex pheromone components during scotophase varied among individual females of the same age (Fig. 2). Repeated-measures ANOVA revealed no significant difference in the titer of the major component (*Z*)*-*11–16:Ald among 0-day-old individuals (*F*(14,70) = 1.33, *P* = 0.212). However, significant inter-individual differences were detected at 1 day (*F*(14,56) = 6.58, *P* < 0.0001), 2 days (*F*(14,70) = 3.72, *P* = 0.0001) and 3 days of age (*F*(14,70) = 2.71, *P* = 0.003) (Fig. 2).

A chi-square test revealed a significant difference in the detection frequency of the major component (*Z*)*-*11–16:Ald across different female ages during the six sampling periods of the scotophase (χ^2^ = 33.49, *P* = 0.002). The percentages of females having (*Z*)*-*11–16:Ald detected in all 6 extractions were 0%, 6.3%, 40%, and 20% for 0-, 1-, 2-, and 3-day-old moths, respectively. Conversely, the percentages of females from which (*Z*)*-*11–16:Ald was not detected in any of the six extractions were 20%, 0%, 0%, and 40% for 0-, 1-, 2-, and 3-day-old females, respectively. The highest frequency of detection (three times per night) was 26.7% in 0-day-old moths, while 37.5% of 1-day-old females had (*Z*)*-*11–16:Ald detected twice per night.

**Fig. 3:**
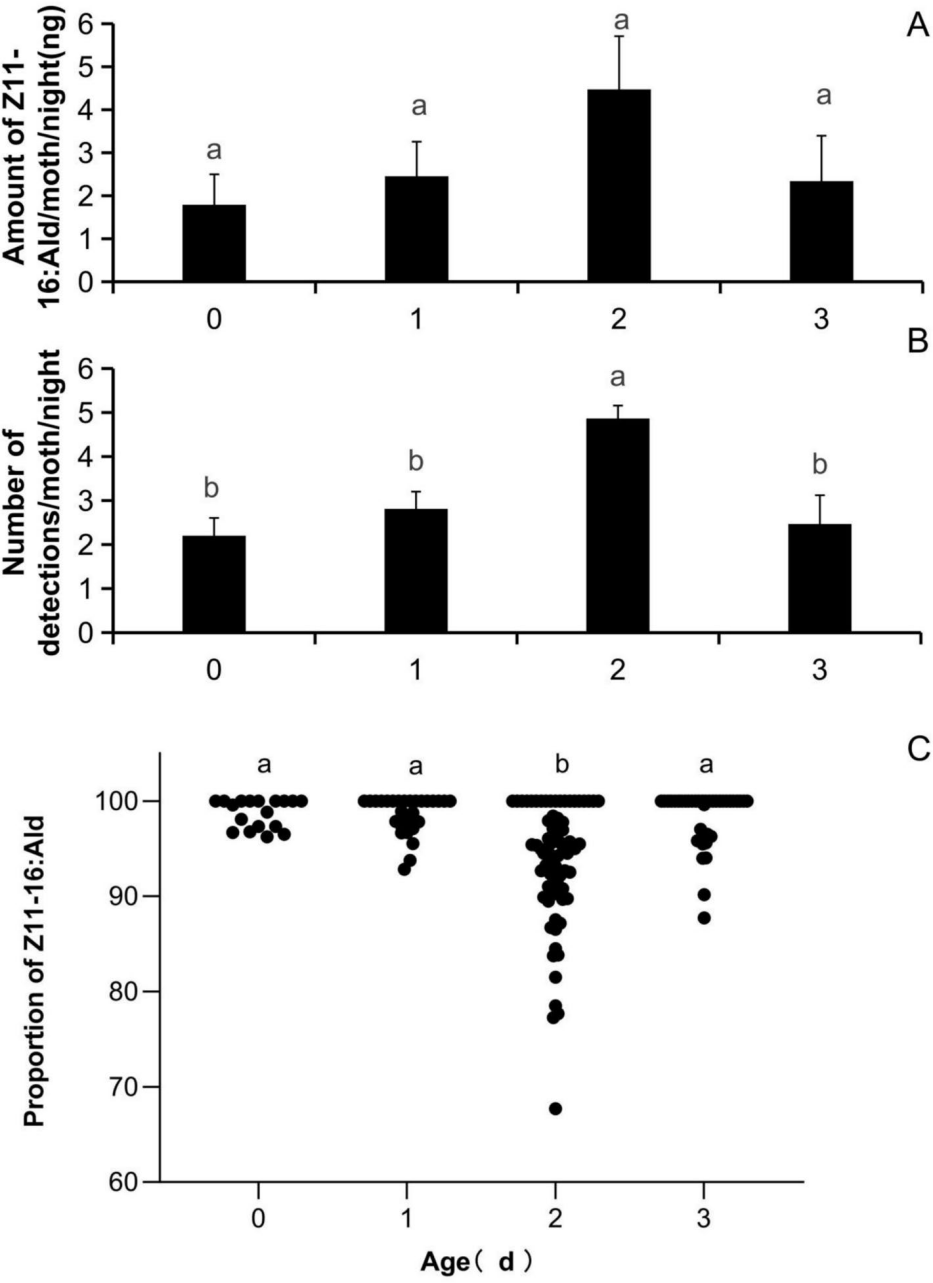
Amount and number of Z11-16:Ald detections from female *C. suppressalis* and the percentage in the mixture of Z11-16:Ald and Z9-16:Ald at different ages. A, Amount of Z11-16:Ald at different ages; B, Number of Z11-16:Ald detections at different ages; C, Percentage of Z11-16:Ald in the mixture of Z11-16:Ald and Z9-16:Ald at different ages. The data in the figure are the mean ± SE. Different letters on the bars indicate significant differences (Duncan's test, one-way ANOVA)

The total amount of (*Z*)*-*11–16:Ald extracted from *C. suppressalis* females during scotophase ranged from 1.8 to 4.5 ng across ages from 0 to 3 days old*.* Although the mean titer was relatively high at 2 days old, there were no significant differences in the amount of (*Z*)*-*11–16:Ald among age groups (*F*(3,57) = 1.45, *P* = 0.239) (Fig. 3A). In contrast, the number of times (*Z*)*-*11–16:Ald was detected per female within a scotophase differed significantly with age, being highest in 2-day-old females (*F*(3,57) = 7.06, *P* < 0.001) (Fig. 3B). The time window for (*Z*)*-*11–16:Ald release was also the longest in the 2-day-old females. Female age significantly altered the released ratio of (*Z*)*-*11- to (*Z*)*-*9–16:Ald in *C. suppressalis* (*F*(3,154) = 14.09, *P* < 0.001) (Fig. 3C). The mean proportion of (*Z*)*-*11–16:Ald relative to the sum of (*Z*)*-*11- and (*Z*)*-*9–16:Ald was significantly lower (93.6 ± 0.8%) in 2-day-old females than in 0-day-old (98.7 ± 0.4%), 1-day-old (98.5 ± 0.4%), and 3-day-old (98.4% ± 0.5%) females (Fig. 3C). Scatter plot analysis confirmed that variation in the percentage of (*Z*)*-*11–16:Ald was the largest among 2-day-old females and smallest among 0-day-old females (Fig. 3C).

### Correlations of pheromone variation

**Fig. 4:**
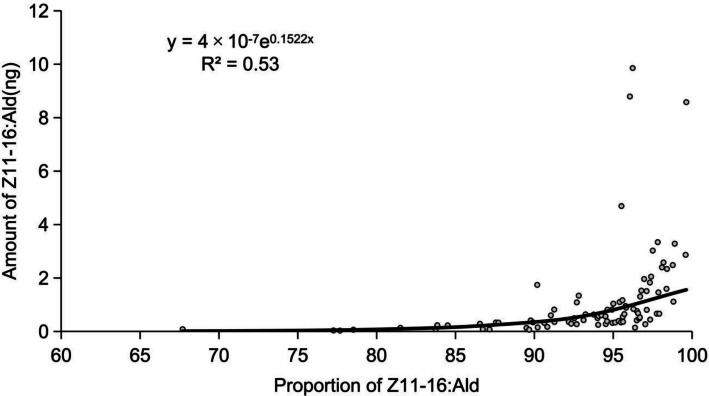
Relationship between the amount of Z11-16:Ald and the percentage of Z11-16:Ald in the mixture of Z11-16:Ald and Z9-16:Ald in female *C. suppressalis*

**Fig. 5:**
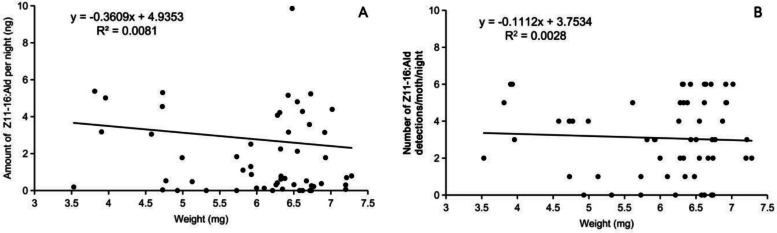
The relationship between body weight and the amount of Z11-16:Ald or number of Z11-16:Ald detections in the sex pheromone of female *C. suppressalis*. A, The relationship between body weight and the amount of Z11-16:Ald; B, The relationship between body weight and number of Z11-16:Ald detections

The percentage of (*Z*)*-*11–16:Ald in the blend was positively correlated with its titer (*r* = 0.37, *P* < 0.001). A linear exponential regression model (*y* = 4 × 10⁻⁷ e^0.152*x*^, *R*^2^ = 0.53) fitted the data, with *x* representing the proportion of (*Z*)*-*11–16:Ald in the blend and y being the titer of (*Z*)*-*11–16:Ald (Fig. 4). However, female body weight showed no significant correlation with either the amount of (*Z*)*-*11–16:Ald (*r* = −0.09, *P* = 0.491) or its detection frequency (*r* = −0.053, *P* = 0.687) (Fig. 5).

### Effect of temperature on sex pheromone release

**Fig. 6:**
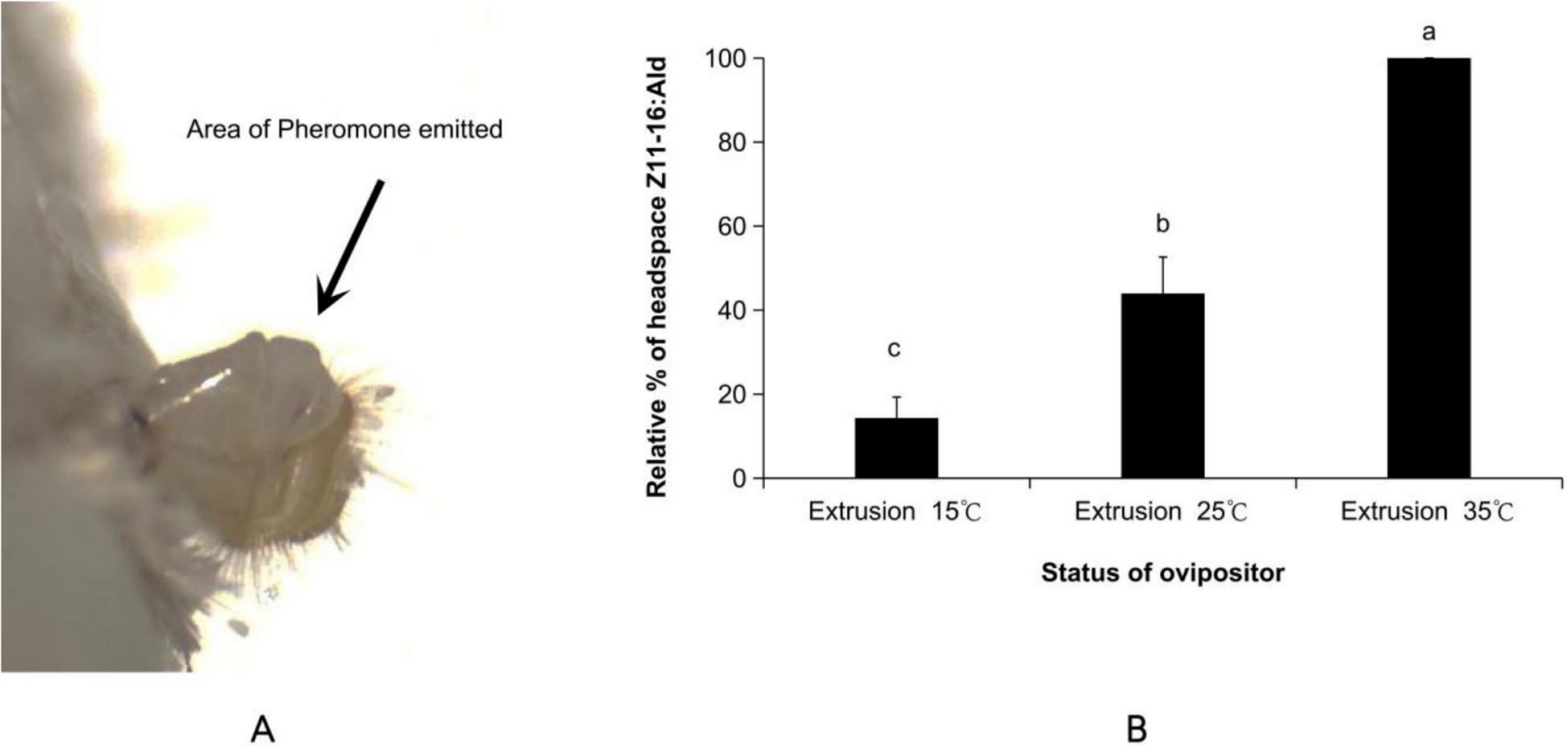
Effect of ambient temperature on pheromone release. The data in the figure are the mean ± SE. Different letters on the bars indicate significant differences (Duncan's test, one-way ANOVA). A. Ovipositor everted; B. Expressed as a percentage of the baseline, the relative titer values for the 15 °C and 25 °C treatments were calculated using the average titer at 35 °C as the reference (defined as 100%)

Temperature significantly influenced the release of the major component (*Z*)*-*11–16:Ald from the pheromone gland surface (*F*(2,15) = 56.56, *P* < 0.001). The amount released increased with temperature. Relative to the release at 35 °C, which was considered 100%, the amounts at 25 °C and 15 °C were 44.0% ± 8.7% and 14.3% ± 5.0%, respectively (Fig. 6).

### Effect of female mating status on sex pheromone release

**Fig. 7:**
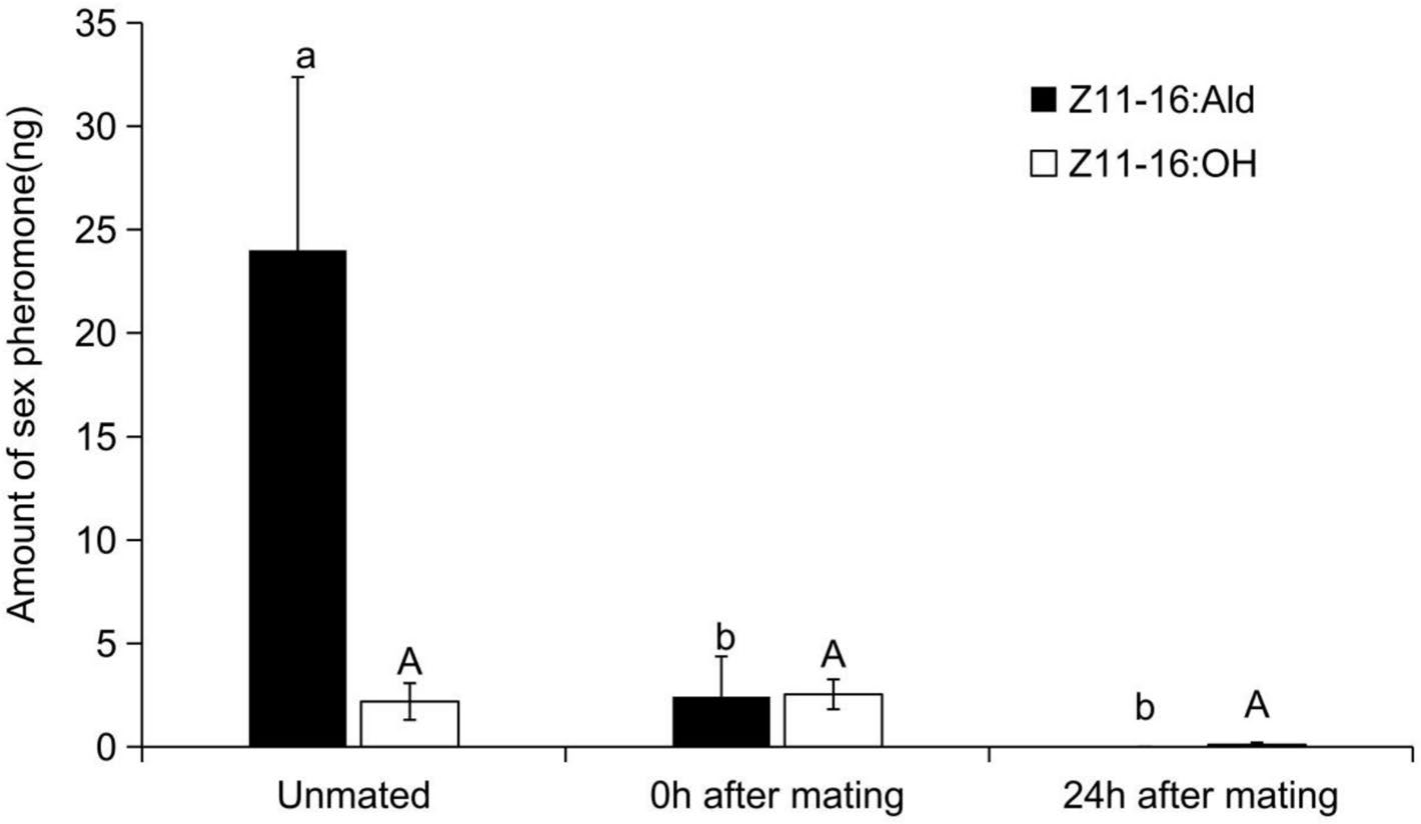
Effect of mating status on the pheromone compound Z11-16:Ald and Z11-16:OH titers. The data in the figure are mean ± SE. Different letters on the bars indicate significant differences (Duncan's test, one-way ANOVA)

A significant effect of mating was observed on the amount of (*Z*)*-*11–16:Ald released (*F* (2,31) = 3.88, *P* = 0.03) (Fig. 7). Post hoc analysis revealed that the quantity from unmated females (24.0 ± 8.4 ng) was significantly higher than that released at 0 h (2.4 ± 2.0 ng) or the non-detectable level at 24 h post mating. There was no significant difference in the amount of (*Z*)*-*11–16:Ald released from the female between 0 and 24 h after mating. Conversely, the release of the minor component (*Z*)*-*11–16:OH was not significantly influenced by mating (*F*(2,31) = 1.781, *P* = 0.185), measuring 2.2 ± 0.9 ng for unmated females, 2.5 ± 0.7 ng at 0 h post-mating, and dropping to trace levels by 24 h post-mating.

### Effect of sex pheromone ratio and titer on male C. suppressalis trapping

**Fig. 8:**
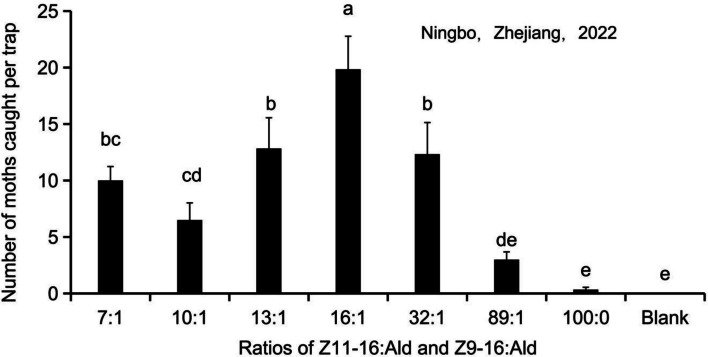
Effect of *C. suppressalis* sex pheromone mixtures composed of different ratios of Z11-16:Ald and Z9-16:Ald on the attractiveness of the males in the field test on May 24-June 7,2022 (Ningbo, Zhejiang). The data in the figure are the mean ± SE. Different letters on the bars indicate significant differences (Duncan's test, one-way ANOVA)

Variation in female sex pheromone blends was primarily reflected in the ratio of (*Z*)*-*11- to (*Z*)*-*9–16:Ald and the total titer. Field experiments demonstrated that different ratios significantly affected the trap catch (*F*(7,40) = 13.59, *P* < 0.001) (Fig. 8). The 16:1 blend was the most effective, trapped 19.8 ± 7.2 moths per trap, which was significantly higher than any other ratio tested. The mean numbers of moth catches for the other blends 7:1, 10:1, 13:1, 32:1, 89:1, and 100:0 (single component) were 10.0 ± 1.2, 6.5 ± 1.5, 12.8 ± 2.7, 13.3 ± 2.8, 3.0 ± 0.7, and 0.3 ± 0.2 moths per trap, respectively. Blank controls caught zero moths.

**Fig. 9:**
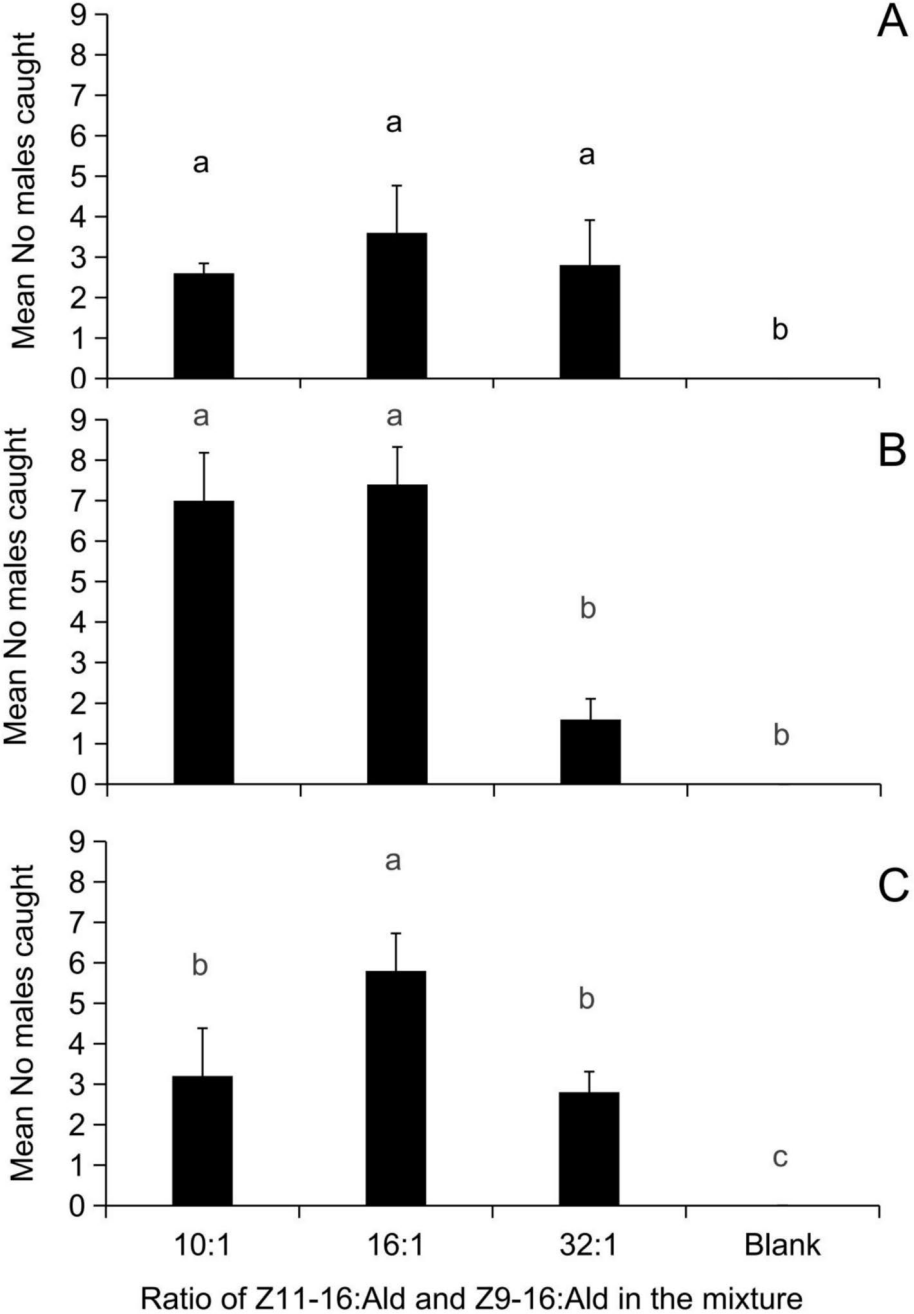
Effect of the *C. suppressalis* sex pheromone mixture with different ratios of *Z*11–16:Ald/*Z*9–16:Ald and different dosages on the attractiveness of the males in the field test on June 12–21, 2022 (Tianyang, Guangxi). The data in the figure are the mean ± SE. Different letters on the bars indicate significant differences (Duncan's test, one-way ANOVA)

The trapping efficacy of three key ratios (10:1, 16:1, 32:1) of (*Z*)*-*11- to (*Z*)*-*9–16 Ald was assessed across three total dosages (760 μg, 1520 μg, and 2280 μg) (Fig. 9). At the 760 μg dosage, no significant difference in capture was found among the three ratios, although each of these ratios caught more moths compared to the control (*F* (3,16) = 11.59, *P* < 0.001). At a dosage of 1520 μg, blends with ratios of 10:1 or 16:1 captured significantly more males than the 32:1 blend (*F*(3,16) = 42.53, *P* < 0.001). At the highest dosage of 2280 μg, the 16:1 blend resulted in a significantly higher moth catch than both the 10:1 and 32:1 blends (*F*(3,16) = 22.25, *P* < 0.001).

### Effect of individual variation on mass trapping in the field

**Fig. 10:**
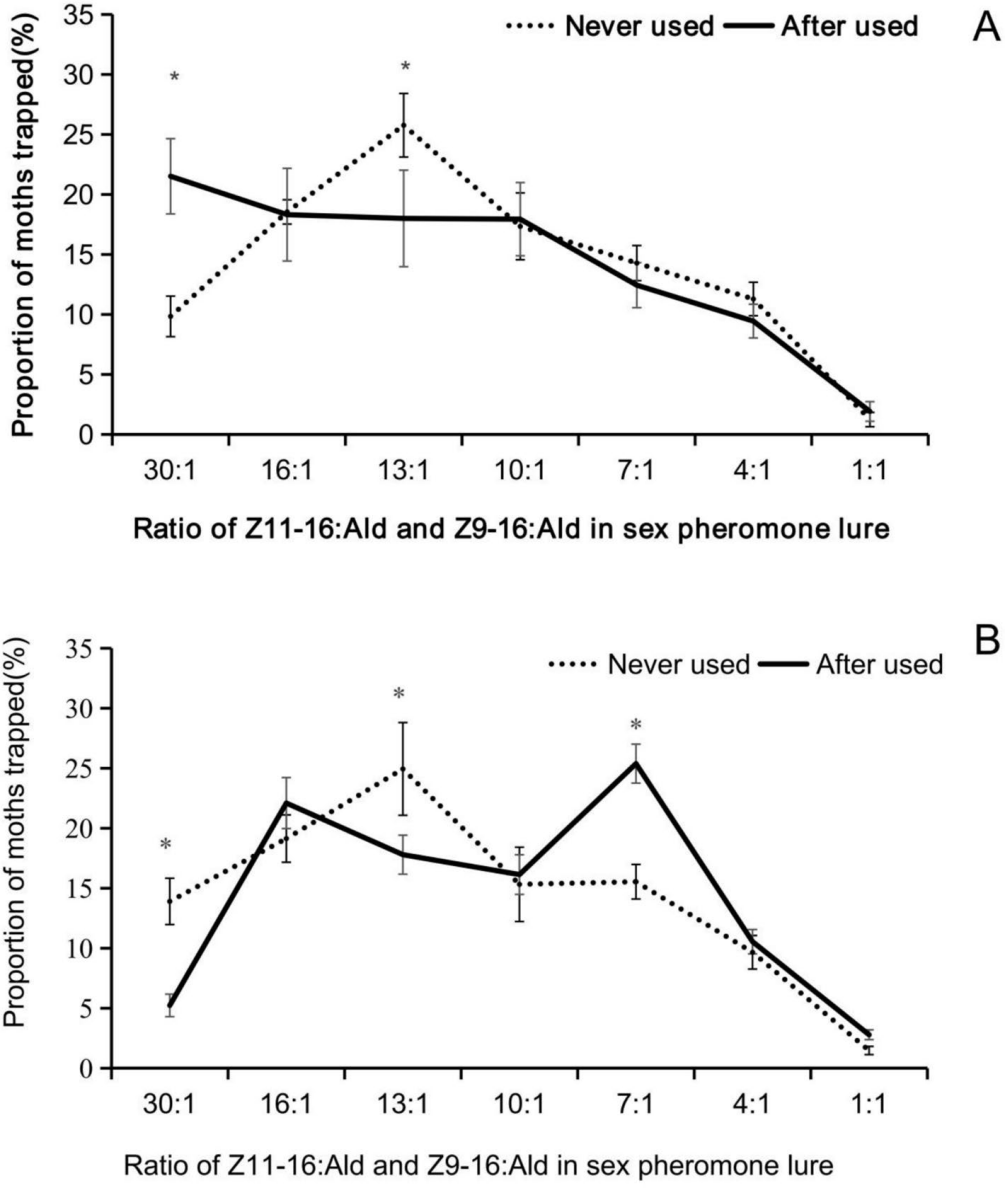
Comparison of a region with long-term mass trapping using a single sex pheromone mixture and a region with no history of sex pheromone trapping. A: Youxian, Hunan; B: Liling, Hunan. Never used (dotted lines) indicates sites with no history of sex pheromone mass trapping, whereas after used (solid lines) refers to sites where mass trapping had been conducted for five consecutive years. The asterisk indicates a significant difference between the two groups (P < 0.05, independent samplest-test)

At the Huangtuling site, the proportion of moths trapped with a 30:1 blend ratio of (*Z*)*-*11–16:Ald to (*Z*)*-*9–16:Ald in the pheromone blend was significantly higher in fields with a history of long-term mass trapping than in control fields (*t* = 4.36, *df* = 9, *P* = 0.021). Conversely, with a 13:1 ratio, the proportion trapped was significantly lower in treatment fields than in controls (*t* = 1.30, *df* = 9, *P* = 0.046) (Fig. 10A). No significant differences were observed between the treatment and control fields for the other tested ratios (13:1: *t* = 1.30, *df* = 9, *P* = 0.196; 10:1: *t* = 1.14, *df* = 9, *P* = 0.899; 7:1: *t* = 0.18, *df* = 9, *P* = 0.514; 4:1: *t* = 0.04, *df* = 9, *P* = 0.425; 1:1: *t* = 0.36, *df* = 9, *P* = 0.574) (Fig. 10A).

At the Sifen site, the proportion of moths trapped with the 30:1 and 13:1 ratios of *Z*11–16:Ald to *Z*9–16:Ald in the sex pheromone blend was significantly lower in fields where sex pheromone mass trapping was previously applied than in their respective control fields (30:1, *t* = 1.75, *df* = 9, *P* = 0.007; 13:1, *t* = 1.14, *df* = 9, *P* = 0.048) (Fig. 10B). Conversely, when the ratio was 7:1, the proportion of moths trapped was significantly higher in the treatment fields than in the control fields (*t* = 0.023, *df* = 9, *P* = 0.003) (Fig. 10B). The proportion of moths trapped was similar with other ratios in the treatment and control fields (16:1: *t* = 0.45, *df* = 9, *P* = 0.038; 10:1: *t* = 2.62, *df* = 9, *P* = 0.847; 4:1: *t* = 1.68, *df* = 9, *P* = 0.665; 1:1: *t* = 0.14, *df* = 9, *P* = 0.052) (Fig. 10B).

## Discussion

While solvent extraction has traditionally been used for pheromone collection, it allows only a single extraction per female moth due to sample destruction. In this study, we employed solid-phase microextraction (SPME) to repeatedly sample the sex pheromone titers of the same individual female *C. suppressalis* across different periods of scotophase and at various ages. Although the pheromone components detected via SPME showed minor differences from those identified through solvent extraction of pheromone glands [22], our approach revealed significant temporal and individual variation in sex pheromone release by female *C. suppressalis*. This variation is critical, as it likely influences female calling behavior in the field and subsequent mating success with conspecific males.

Insect sex pheromone variation, arising from individual differences within populations [33], can manifest as changes in the ratio of blend components or in the chemical structure and composition of the pheromone itself. The former involves shifts in the proportions of the same components, while the latter indicates the presence of entirely different compounds across populations. Such variation in female pheromone production is often mirrored by individual and geographic differences in the male olfactory response [15, 18]. Variation in blend ratios is a common feature of insect olfaction systems, as seen in *C. suppressalis* [23] and *Phthorimaea operculella* [34]. A classic example is *Ostrinia nubilalis*, which has two distinct biotypes in North Carolina defined by opposite ratios of (*cis*)−11- and (*trans*)−11- tetradecene acetate [35]. Similarly, rice and maize strains of *Spodoptera frugiperda* exhibit different ratios of key pheromone compounds [36]. In some cases, variation involves distinct chemical structures and compositions. *Mythimna separata* populations use one of two pheromone types, either (*Z*)*-*11–16:Ald alone or a blend of (*Z*)*-*11–16:OAc and (*Z*)*-*11–16:OH [18, 37]. In *Cnaphalocrocis medinalis*, Japanese and Chinese populations share a four-component blend [38], whereas Indian and the Philippine populations produce a different blend [39]. For *C. suppressalis*, components such as (*Z*)*-*11–16:OH and 16:Ald ensure species specificity without directly enhancing attraction (Chen et al., 2018). (*Z*)*-*13–18:Ald is an essential, but moderate variation in its amount does not significantly alter attractiveness (Chen et al., 2018). Temperature influences calling behavior and pheromone release [40], which may affect female competitiveness in the field. Consequently, this study focused on variation in the (*Z*)*-*11-/(*Z*)*-*9–16:Ald ratio, which is directly linked to male attraction[22, 23]. A positive correlation was found between this ratio and pheromone titer, a finding crucial for optimizing blends in field trapping. Field experiments confirmed that a 16:1 ratio was optimal, with deviations reducing catches and the absence of (*Z*)*-*9–16:Ald eliminating attraction entirely.

In *C. suppressalis,* adult age governs key reproductive behaviors, significantly altering the proportion of females that call and the timing of sex pheromone emission. At 0 and 1 day of age, the pheromone release duration was short, and the titer was low. By 2 days of age, the release duration increased significantly, although the absolute titer did not. Additionally, two-day-old females exhibit a broad range of pheromone blend ratios, which may attract a wider range of males with divergent response profiles. This suggests a trade-off between the metabolic cost of sex pheromone release (low concentration, short duration) and reproductive fitness, with costs increasing as aging continues and investment in pheromone release persists [5, 41]. Since most females mate only once, it is adaptive for pheromone secretion to decline rapidly post-mating. It is noteworthy that (*Z*)*-*11–16:OH remains present in many pre- and post- mating extracts (Fig. S1). As a precursor to (*Z*)*-*11–16:Ald, this indicates either a rapid cessation of aldehyde synthesis or a time lag in the final biosynthetic step from (*Z*)*-*11–16:OH to (*Z*)*-*11–16:Ald, warranting further investigation into post-mating male olfactory responses[42]. The synthetic pathways for (*Z*)*-*11–16:Ald and (*Z*)*-*9–16:Ald differ, involving Δ9 and Δ11 desaturases [43]. Therefore, factors influencing these pathways can alter the final blend ratio. Additionally, we also note that individual females exhibited a relatively short calling period in our experiment, likely due to artificial gland exposure; thus, under natural conditions, this period might be even shorter. Furthermore, no relationship was found between body weight and pheromone release in *C. suppressalis,* a result inconsistent with findings in *S. littoralis* [44] and *Lobesia botrana* [45], where larger individuals produced high pheromone titers and were preferred by males.

Sex pheromones, as premating signals, are typically stable and innate. However, their synthesis and release are metabolically costly [41]. This cost creates a covariance between signal and fitness under natural selection, while genetic variation is maintained through sexual selection [5]. Males must co-evolve to adapt their olfactory systems to variations in female pheromones. For instance, pheromone production is linked to hemolymph trehalose levels in *Heliothis virescens*, allowing males to assess female nutritional status through pheromone signaling [46]. Signal accuracy is encoded in the quantitative ratio of components [46]. This inherent variation poses a significant challenge for applied pheromone technology in field trapping, as a single blend ratio may not attract all males in a population, given that some males may be adapted to other ratios.

Natural variation exists in both the sex pheromones produced by female *C. suppressalis* and the olfactory responses of males. This variability underscores the need to assess the potential evolutionary consequences of long-term, large-scale application of a single pheromone ratio on target pest behavior. Here, we compared changes in male olfactory responses to different pheromone blends after five consecutive years of mass trapping using a 10:1 ((*Z*)*-*11-/(*Z*)*-*9–16:Ald) formulation at two sites, Huangtuling and Sifen. Our findings indicate that prolonged selection pressure led to directional behavioral adaptations in both populations toward ratios not used in the field. Notably, these changes reflect fine-tuning of the response spectrum rather than simple loss of attraction. In Huangtuling, populations showed an enhanced response to a 30:1 ratio but a weakened response to 13:1. In Sifen, responses decreased for 30:1 and 13:1 but increased for 7:1. These results strongly suggest that sustained exposure to a fixed ratio altered the pre-mating communication system of *C. suppressalis*. The divergent patterns between populations further indicate geographic specificity in adaptive evolution. These trajectories may stem from initial genetic structure or local ecological factors, such as host plant varieties, temperature, and pesticide history, influencing selection direction. Notably, the complete pheromone blend includes additional components beyond (*Z*)*-*11–16:Ald and (*Z*)*-*9–16:Ald, such as (*Z*)*-*13–18:Ald, (*Z*)*-*11–16:OH, and 16:Ald. Although the (*Z*)*-*11-/(*Z*)*-*9–16:Ald ratio is behaviorally relevant, its categorization can be somewhat arbitrary.

Olfactory adaptation, driven by standing variation in female pheromone production, can lead to divergent olfactory responses among male moths, manifesting as distinct preferences for specific pheromone ratios and dosages. These behavioral shifts may arise from altered sensitivity of olfactory receptor neurons or from central nervous system integration and remodeling. Even a single amino acid mutation in an olfactory receptor can contribute to such variation [47]. After consecutive years of mass trapping with a fixed-ratio synthetic pheromone, male moths initially sensitive to that ratio gradually lost preference, while the proportion favoring other ratios increased. From a trapping perspective, this suggests that *C. suppressalis* males have developed behavioral resistance to the sex pheromone. Long-term exposure to a single blend may progressively eliminate males preferring the deployed ratio, thereby favoring those with different sensory sensitivities. The earliest documented case of such resistance comes from mating disruption studies in pink bollworms [48]. These findings carry important implications for pest management. Consequently, pheromone-based mass trapping strategies must anticipate such rapid evolutionary responses and preserve long-term effectiveness by rotating pheromone blends or integrating them with complementary approaches.

A key limitation of this study is its focus on behavioral outputs in the field. Future work should explore how genes such as Δ9 desaturase regulate the Z11-/Z9-16:Ald ratio. Investigating the genetic basis of pheromone biosynthesis and male olfactory receptors could reveal whether standing variation underlies differential adaptive differences among populations. Additionally, the optimal blend composition often depends on the total dosage, necessitating extensive field trials. Sustained mass trapping with a fixed blend may impose selective pressure on genes involved in sex pheromone production or olfactory perception, reshaping the genetic architecture of this multicomponent signal [49]. Over time, this could reduce trapping efficiency, compromising both monitoring reliability and control efficacy.

## Supplementary Information

Supplementary Material 1: Fig. S1 GC-MS showed that (Z)-11-16:OH was still present in some of the pre- and post-mating female moth extracts.

## Data Availability

Not applicable.
