# Supplementary figures and images for: Temporal variation in sex pheromone release from individual Chilo suppressalis (Lepidoptera: Crambidae) females and maximization of male trapping
Source: Crop Health. 2026 Apr 1;4(1):9. doi: 10.1007/s44297-026-00071-w (PMC13038809; doi:10.1007/s44297-026-00071-w)

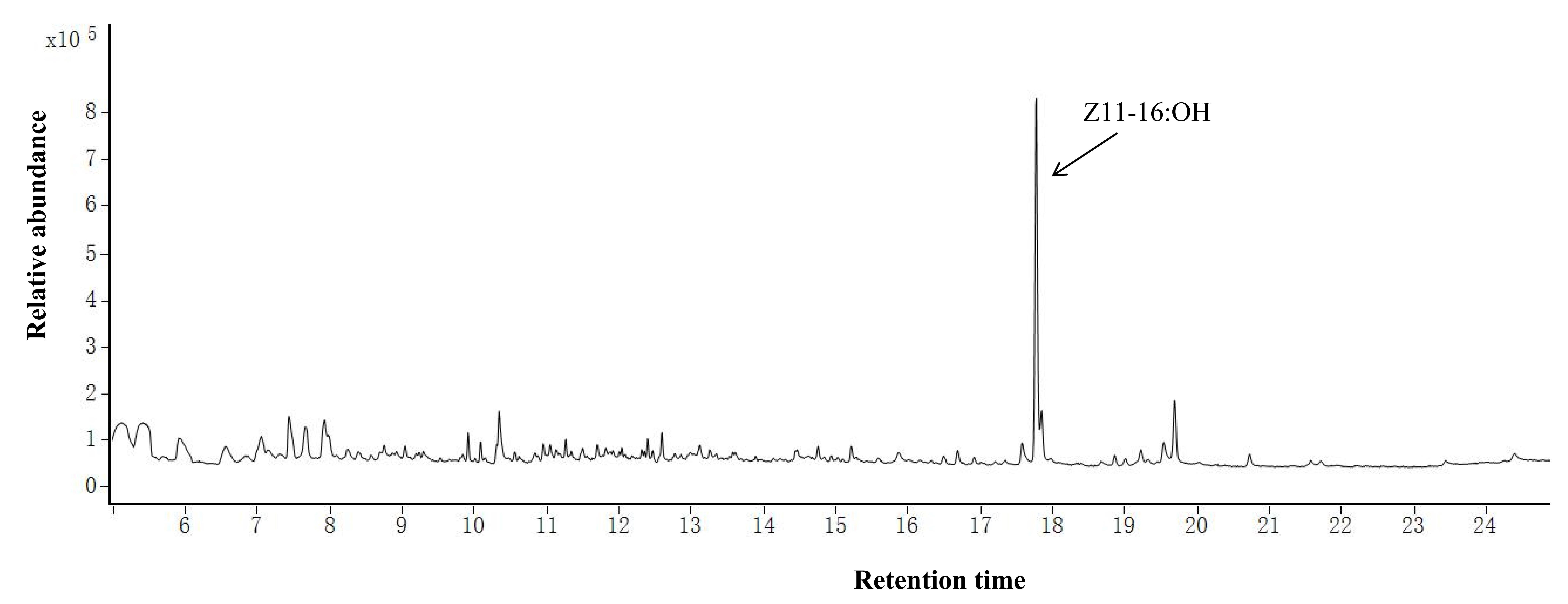

Supplement: Supplementary file 1 — Supplementary Material 1: Fig. S1 GC-MS showed that (Z)-11-16:OH was still present in some of the pre- and post-mating female moth extracts. [file 44297_2026_71_MOESM1_ESM.jpg]
